# Interleukin-18: A Novel Participant in the Occurrence, Development, and Drug Therapy of Obliterative Bronchiolitis Postlung Transplantation

**DOI:** 10.1155/2021/5586312

**Published:** 2021-07-27

**Authors:** Ping Shu, Wei Zhang, Yanfei Zhang, Yanfeng Zhao, Yuping Li, Xiaoqing Zhang

**Affiliations:** ^1^Department of Pharmacy, Shanghai Pulmonary Hospital, Tongji University School of Medicine, Shanghai 200433, China; ^2^Department of Pharmacy, The International Peace Maternity and Child Health Hospital, School of Medicine, Shanghai Jiao Tong University, Shanghai 200030, China; ^3^Department of Thoracic Surgery, Shanghai Pulmonary Hospital, Tongji University School of Medicine, Shanghai 200433, China

## Abstract

**Background:**

Obliterative bronchiolitis (OB) was a main cause of deterioration of long-term prognosis in lung transplant recipients after the first posttransplant year. Proinflammatory cytokine interleukin-18 (IL-18) strengthened both the natural immunity and acquired immunity and played an important role in organ transplantation. The roles of IL-18 in the occurrence, development, and drug treatment of OB remained unclear.

**Methods:**

Small interfering RNA (siRNA) against mouse IL-18 (siRNA-IL-18) was used to silence IL-18 expression. Mouse heterotopic tracheal transplantation model was used to simulate OB. Recipient mice were divided into 5 groups (*n* = 12) according to donor mouse strains and drug treatment: isograft group, allograft group, allograft+tacrolimus group, allograft+azithromycin group, and allograft+tacrolimus+azithromycin group. The luminal obliteration rates were pathological evaluation. Expressions of cytokines and MMPs were detected by real-time PCR, western blot, and enzyme chain immunosorbent assay (ELISA).

**Results:**

The luminal obliteration rates of IL-18 of the siRNA-IL-18 group were significantly lower than those of the negative control group (*p* < 0.0001) and the blank control group (*p* = 0.0002). mRNA expressions of IFN-*γ*, EMMPRIN, MMP-8, and MMP-9 of the siRNA-IL-18 group were significantly lower than those of the negative and blank control groups. No tracheal occlusion occurred in grafts of the isograft group. The rates of tracheal occlusion of the allograft group, allograft+tacrolimus group, allograft+azithromycin group, and allograft+tacrolimus+azithromycin group were 72.17 ± 4.66%, 40.33 ± 3.00%, 38.50 ± 2.08%, and 23.33 ± 3.24%, respectively. There were significant differences between the 4 groups (*p* < 0.001). Serum protein expressions of IL-17 (*p* = 0.0017), IL-18 (*p* = 0.0036), IFN-*γ* (*p* = 0.0102), and MMP-9 (*p* = 0.0194) were significantly decreased in the allograft+tacrolimus+azithromycin group compared to the allograft group.

**Conclusions:**

IL-18 could be a novel molecular involved in the occurrence, development, and drug treatment of OB.

## 1. Introduction

Lung transplantation is the only effective therapy to patients with end-stage lung diseases. One-year survival rate of lung transplant recipients had improved remarkably from less than 45% to almost 90% over the past 40 years, which was due to advances in surgical technique, prevention, and treatment of postoperative infection, immunosuppressive regimen, and perioperative nursing [1]. However, lung transplant had a poor long-term prognosis with median survival time after transplantation limited to 6.5 years. Chronic lung allograft dysfunction (CLAD) was the main cause of death in lung transplant recipients after the first posttransplant year. Obliterative bronchiolitis (OB) was the common CLAD phenotype characterized by small airway luminal narrowing attributed to external compression by the inflammation and fibrosis process [2]. Bronchiolitis occlusive syndrome (BOS), the clinical presentations of OB, includes progressive occlusion of the bronchioles, small airway dysfunction, and respiratory block. The 5-year survival rate of lung transplantation patients with BOS was only 30-40%, which is about 30% less than that of lung transplantation patients without BOS [3].

There were 3 stages in the process of OB occurrence and development: the initial step was pulmonary epithelial cell damage caused by acute rejection, lymphocytic bronchiolitis, cytomegalovirus infection, and gastroesophageal reflux; then, inflammatory cascade was triggered, and a variety of cytokines and chemokines were upregulated; after the repetitive damage by inflammatory response, tissue remodeling of bronchiole occurred which resulted in fibrosis and obliteration of the airway lumen [4–6].

Chronic rejection was the main pathogenesis of OB. In chronic graft rejection lung transplant patients, regulatory and effector cells clearly demonstrated different pathways of activation. Understanding the complex crosstalk between T cells and B cells, both in at alveolar and peripheral levels, was necessary to improve the biology of these cells during rejection after lung transplant. The balance of T cells and regulatory cells, including Tregs and new phenotypes such as Bregs, can offer insights into allograft rejection [7]. Also, a variety of cytokines were involved in the pathogenesis. Proinflammatory cytokine interleukin-18 (IL-18) strengthened both the natural immunity and acquired immunity and played an important role in organ transplantation. IL-18 stimulated lymphocytes to produce the IFN-*γ* and regulate macrophage activity, thereby increasing the expression of proinflammatory cytokines including IL-1*β*, IL-6, CCL4, CXCL2, and CCL2. It was found that IL-18 signaling pathway contributed to vascular transplantation, ischemmia/reperfusion, acute kidney injury, and acute rejection of kidney/liver/heart transplantation. Neutralizing IL-18 by its inhibitor IL-18 binding protein could efficiently suppress the production of injury-associated cytokines such as IL-6, TNF-*α*, IFN-*γ*, CXCL10 (IFN-*γ*-inducible protein 10), and CX3CL1 (fractalkine) and improve allograft function. Alteration of IL-18 levels was suggested as a biomarker for predicting ongoing allograft outcome [8–10]. The roles of IL-18 in the OB occurrence and development remained unclear.

Cytokines were involved in injury mechanism of pulmonary epithelial cells during the development of OB. A review showed that genetic polymorphisms of cytokine genes had been linked to the susceptibility to develop BOS after lung transplantation. A single nucleotide polymorphism (SNP) chip for cytokine genes might be a promising approach for predicting the risk of BOS and could help clinicians to establish individualized management for the prevention and treatment of BOS [11]. Interleukin-17 (IL-17) was an OB-related molecule confirmed by many studies [12–14]. The deficiency of IL-17 suppressed M1 macrophage polarization and function. Macrophage infiltration had been turned out to be the main reason for rejection. The pathological damage degree of IL-17-/- mice was lighter than that of WT recipients in murine heterotopic tracheal transplantation models [12]. In another study, a highly reproducible preclinical model of OB, transplantation of minor histoincompatible lungs from C57BL/10 mice into C57BL/6 mice, was used. The mRNA transcripts and serum protein levels of IL-17A, a member of cytokine family, were increased only in mice that developed OB. Neutralizing IL-17 could prevent OB [13]. IFN-*γ* was closely related to chronic rejection. Polymorphisms at position +874 of the IFN-*γ* gene significantly increase the risk for BOS development after lung transplantation [15]. IFN-*γ* involved in the immune response to tissue-restricted self-antigen- (SAg-) induced airway inflammation and fibrosis following lung transplantation [16, 17].

Matrix metalloproteinases (MMPs) participated in tissue remodeling and promoted the formation of OB. Levels of MMP-8 and MMP-9 were higher in BOS. Allografts transplanted into MMP-9-/- recipients did not develop obliterative airway disease (OAD) [18–20]. It is essential to determine the mRNA and protein expression of MMPs to explore the role of novel participant of OB.

At present, no satisfactory treatment was available for OB. Tacrolimus was one kind of widely used immunosuppressive agents after lung transplantation. Tacrolimus could inhibit OAD in a dose-dependent manner, and that early administration arrested OAD progression, and that late administration slowed OAD progression [21]. Azithromycin was another alternative drug for OB. A randomized controlled trial showed that azithromycin therapy improved FEV1 in patients with BOS and appeared superior to placebo [22]. The effect of combination of tacrolimus and azithromycin on BOS and OB-related molecule was not yet known.

In this study, the roles of IL-18 in occurrence, development, and drug therapy of OB were explored using RNA interference, animal model, and pathological evaluation. Known OB-related molecules, such as IL-17, IFN-*γ*, MMP-8, and MMP-9, were incorporated in this research. We aimed to provide novel molecule and its mechanism for OB.

## 2. Materials and Methods

### 2.1. Animals

Male C57BL/6 and BALB/c mice bred under specific pathogen-free (SPF) conditions were purchased from Shanghai SLAC Laboratory Animal Co. Ltd (Shanghai, China). At the time of the experiments, these mice weighed at 20 ± 2 g. All animal experiments complied with the guidelines of the Principles of Laboratory Animal Care (NIH publication Vol 25, No. 28 revised 1996) and the Shanghai Pulmonary Hospital Animal Care and Use Committee Guidelines.

### 2.2. Model Building of Mouse Heterotopic Tracheal Transplantation

Mouse heterotopic tracheal transplantation was a recognized animal model of simulating the pathophysiology of OB. In isograft model, trachea of donor BALB/c mice were transplanted to BALC/c recipients, while trachea of donor C57BL/6 mice were transplanted to BALB/c recipients in allograft model. The mice were under the anesthesia with the injection of Ketamine (100 mg/kg) in abdominal cavity during operation process. Entire trachea, from the inferior edge of cricoid cartilage to bifurcation of trachea, was exposed through a median incision in the neck. Donor trachea were harvested through the process included resecting, flushing, and preserving with cold sterile saline at 4°C. Surgical process of recipient included the following: a small incision was made in the dorsal suprascapular area; subcutaneous tunnel was built by blunt separation; donor trachea was placed in the tunnel; incision was sutured with interrupted 7-0 Vicryl.

Recipient mice were divided into 5 groups according to donor mouse strains and drug treatment, and each group contained 12 mice. Isograft group: trachea of BALB/c donors were transplanted to BALB/c recipients; allograft group: trachea of C57BL/6 donors were transplanted to BALB/c recipients; allograft+tacrolimus group: trachea of C57BL/6 donors were transplanted to BALB/c recipients, and the recipients received tacrolimus (1 mg/kg/day) by intragastric administration twice a day; allograft+azithromycin group: trachea of C57BL/6 donors were transplanted to BALB/c recipients, and the recipients received azithromycin (15 mg/kg/day) by intragastric administration once every 3 days; allograft+tacrolimus+azithromycin group: trachea of C57BL/6 donors were transplanted to BALB/c recipients, and the recipients received tacrolimus (1 mg/kg/day, twice a day) and azithromycin (15 mg/kg/day, once every 3 days) by intragastric administration. Treatment of tacrolimus and azithromycin was performed from the first to the 27th day after the operation. All groups were dissected to collect serum and peripheral blood for ELISA detection; the grafts were harvested from recipient mice on day 28 after transplantation for histopathology and western blot assay.

### 2.3. Plasmid Construction and Transfection of siRNA-IL-18

Small interfering RNA (siRNA) against mouse IL-18 (siRNA-IL-18) was synthesized by Sangon Biotech (Shanghai) Co., Ltd. and used to silence IL-18 expression. The sequence of siRNA targeting mouse IL-18 was CCCTCTCCTGTAAGAACAA. Cells were transfected with siRNA-IL-18 using Lipofectamine 2000 Reagent (Invitrogen) according to the manufacturer's protocol.

Lentivirus-mediated siRNA-IL-18 vector (108 pfu/15 *μ*l) was injected into subcutaneous tunnel of allograft mouse heterotopic tracheal transplantation by micropuncture apparatus on the postoperative days 1, 14, and 21. Blank Lentivirus vector (108 pfu/15 *μ*l) and phosphate buffer solution (PBS, 15 *μ*l) were used as negative control group and blank control group. Each group underwent with 10 repetitions.

### 2.4. Assessment of the Obliteration Ratio of Tracheal Grafts

Tracheal grafts were resected from recipient mice, immediately fixed in 4% formalin for 24 hours at room temperature, and then embedded in paraffin. Paraffin-embedded blocks of tracheal graft tissues were cut into 4 *μ*m sections. The changes of tracheal structure and inflammatory infiltration were observed using hematoxylin and eosin (H&E) stain under optical microscope. The ratio of occlusion area to cross section of trachea was the luminal obliteration rate. All histologic evaluations were performed by two individual observers in a blinded manner.

### 2.5. mRNA Expression of Cytokines and MMPs Detected by Real-Time PCR

Total RNA was isolated from mouse tracheal epithelium cells and donor trachea tissues using TRIzol Reagent (Invitrogen, Carlsbad, CA, USA) according to the manufacturer's protocol. The first strand cDNA was synthesized from 1 *μ*g of total RNA as the template using the RevertAid First Strand cDNA Synthesis Kit (MBI Fermentas, Vilnius, Lithuania). Real-time PCR of IL-18, IFN-*γ*, EMMPRIN, MMP-8, and MMP-9 was performed on an Applied Biosystems™ ViiA™ real-time PCR system (Thermo Fisher Scientific, Waltham, MA, USA) according to the manufacturer's instructions. GAPDH was used as endogenous control.

### 2.6. Protein Expression of Cytokines and MMPs Detected by Western Blot Assay

Western blot was performed to assess IL-18 protein expression in mouse tracheal epithelium cells and the protein expression of IL-17, IL-18, IFN-*γ*, MMP-8, and MMP-9 in tracheal graft of different groups of mouse heterotopic tracheal transplantation model. Whole protein was extracted using radioimmunoprecipitation assay (RIPA) (Roche, Germany). Protein concentration was measured by Pierce BCA Protein Assay Kit (Thermo Scientific, USA). The proteins separated by SDS-PAGE were electrophoretically transferred onto PDVF membranes (Bio-Rad, USA). IFN-*γ* antibody was purchased from biorbyt (Cambridge, Cambridgeshire, United Kingdom). Primary antibodies against IL-17, IL-18, MMP-8, and MMP-9 were purchased from Abcam (Cambridge, MA, USA). Actin was used as loading control. Secondary antibody was DyLight 800 goat anti-rabbit IgG (H+L) (KPL, USA). Odyssey infrared imaging system (LI-COR Biosciences, USA) was used for quantitative western blots.

### 2.7. Detection of Serum Concentration of Cytokines and MMPs

The serum concentration of cytokines (IL-17, IL-18, and IFN-*γ*) and MMPs (MMP-8, MMP-9, and EMMPRIN) was detected by enzyme chain immunosorbent assay (ELISA). The operation procedures are strictly in accordance with the kit instructions for testing (American R&D company, Minneapolis, USA). The absorbance (*A*) value of each hole was measured at 450 nm wavelength and zero with a blank hole. The standard curve was made and found out the content of blood sample from the standard curve.

### 2.8. Statistical Analysis

The SPSS19.0 statistical software (IBM Corporation, Armonk, NY, USA) and GraphPad Prism 8 (GraphPad Software, San Diego, CA, USA) were used for data analysis and chart making. All measurement data are presented as means ± SEM. Statistical analysis was performed using a one-way ANOVA followed by a Bonferroni-Holm correction and *t*-test. A *p* value of <0.05 was regarded as statistically significant.

## 3. Results

### 3.1. Effect of siRNA-IL-18 on mRNA and Protein Expression of IL-18 of Tracheal Epithelium Cells

**Figure 1:**
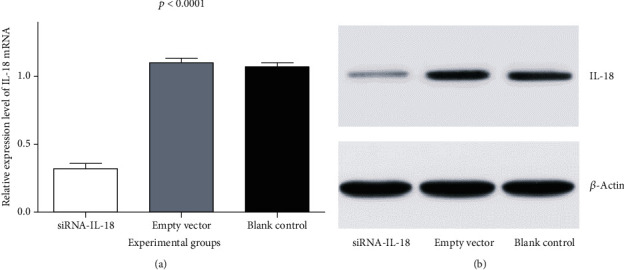
Effects of siRNA-IL-18 on IL-18 mRNA and protein of tracheal epithelium cells. (a) Effects of siRNA-IL-18 on IL-18 mRNA of tracheal epithelium cells and (b) effects of siRNA-IL-18 on IL-18 protein of tracheal epithelium cells.

Tracheal epithelium cells of allograft mouse heterotopic tracheal transplantation were treated by siRNA-IL-18 (experiment group), blank vector (negative control group), and phosphate buffer solution (PBS, blank control group). Each group underwent with 10 repetitions. Relative expressions of IL-18 of the siRNA-IL-18 group, negative control group, and blank control group were 0.32 ± 0.04, 1.10 ± 0.03, and 1.07 ± 0.03, respectively. IL-18 mRNA of the siRNA-IL-18 group was significantly lower than that of the negative control group and blank control group (*p* < 0.0001). IL-18 mRNA of the negative control group and blank control group was not significantly different (*p* = 0.4721) (Figure 1(a)). Western blot analysis showed that IL-18 protein of the siRNA-IL-18 group was lower than that of the negative control group and blank control group (Figure 1(b)).

### 3.2. Effect of siRNA-IL-18 on Rates of Tracheal Occlusion in Mouse Heterotopic Tracheal Transplantation Model

**Figure 2:**
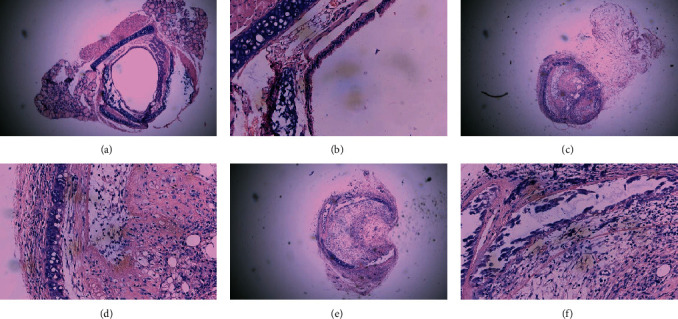
Effects of siRNA-IL-18 on the donor tracheal occlusion rates of mouse heterotopic tracheal transplantation model (hematoxylin-eosin staining). (a) siRNA-IL-18 group (×40), (b) siRNA-IL-18 group (×400), (c) empty vector group (×40), (d) empty vector group (×400), (e) blank control group (×40), and (f) blank control group (×400).

The luminal obliteration rates of IL-18 of the siRNA-IL-18 group, negative control group, and blank control group were 33.30 ± 10.74%, 64.50 ± 12.78%, and 67.30 ± 19.22%, respectively (Figure 2). There was no significant difference between the negative control group and blank control group (*p* = 0.7297). The luminal obliteration rate of IL-18 of the siRNA-IL-18 group was significantly lower than the negative control group (*p* < 0.0001) and blank control group (*p* = 0.0002).

### 3.3. Effect of siRNA-IL-18 on mRNA Expression of IFN-*γ*, EMMPRIN, MMP-8, and MMP-9

**Figure 3:**
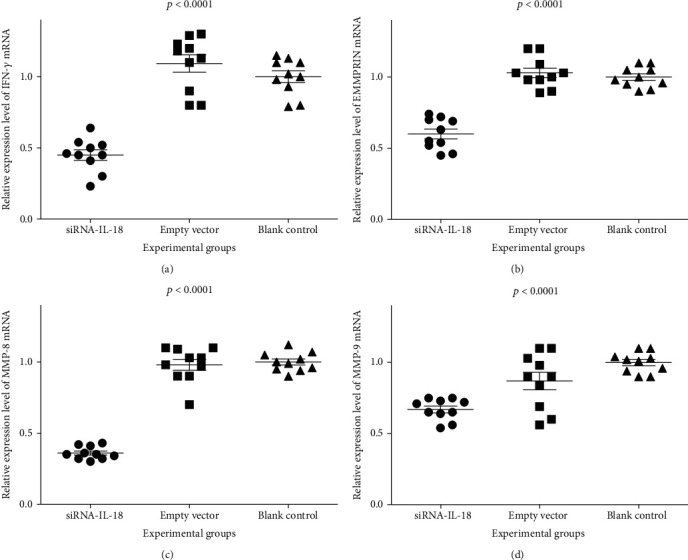
Effects of siRNA-IL-18 on mRNA expression of IFN-*γ*, EMMPRIN, MMP-8, and MMP-9 of tracheal epithelium cells treated by siRNA-IL-18 group, empty vector (negative control), and PBS (blank control). (a) Expression difference of IFN-*γ* mRNA among three cell groups. (b) Expression difference of EMMPRIN mRNA among three cell groups. (c) Expression difference of MMP-8 mRNA among three cell groups. (d) Expression difference of MMP-9 mRNA among three cell groups.

Relative IFN-*γ* mRNA expressions of the siRNA-IL-18 group, negative control group, and blank control group were 0.45 ± 0.04, 1.09 ± 0.06, and 1.00 ± 0.04, respectively. Relative EMMPRIN mRNA expressions of the siRNA-IL-18 group, negative control group, and blank control group were 0.60 ± 0.04, 1.03 ± 0.03, and 1.00 ± 0.02, respectively. Relative MMP-8 mRNA expressions of the siRNA-IL-18 group, negative control group, and blank control group were 0.36 ± 0.01, 0.98 ± 0.04, and 1.00 ± 0.02, respectively. Relative MMP-9 mRNA expressions of the siRNA-IL-18 group, negative control group, and blank control group were 0.67 ± 0.02, 0.87 ± 0.06, and 1.00 ± 0.02, respectively. mRNA expressions of IFN-*γ*, EMMPRIN, MMP-8, and MMP-9 of the siRNA-IL-18 group were significantly lower than those of the negative and blank control groups (Figure 3).

### 3.4. The Rate of Tracheal Occlusion of Different Groups of Mouse Heterotopic Tracheal Transplantation Model

**Figure 4:**
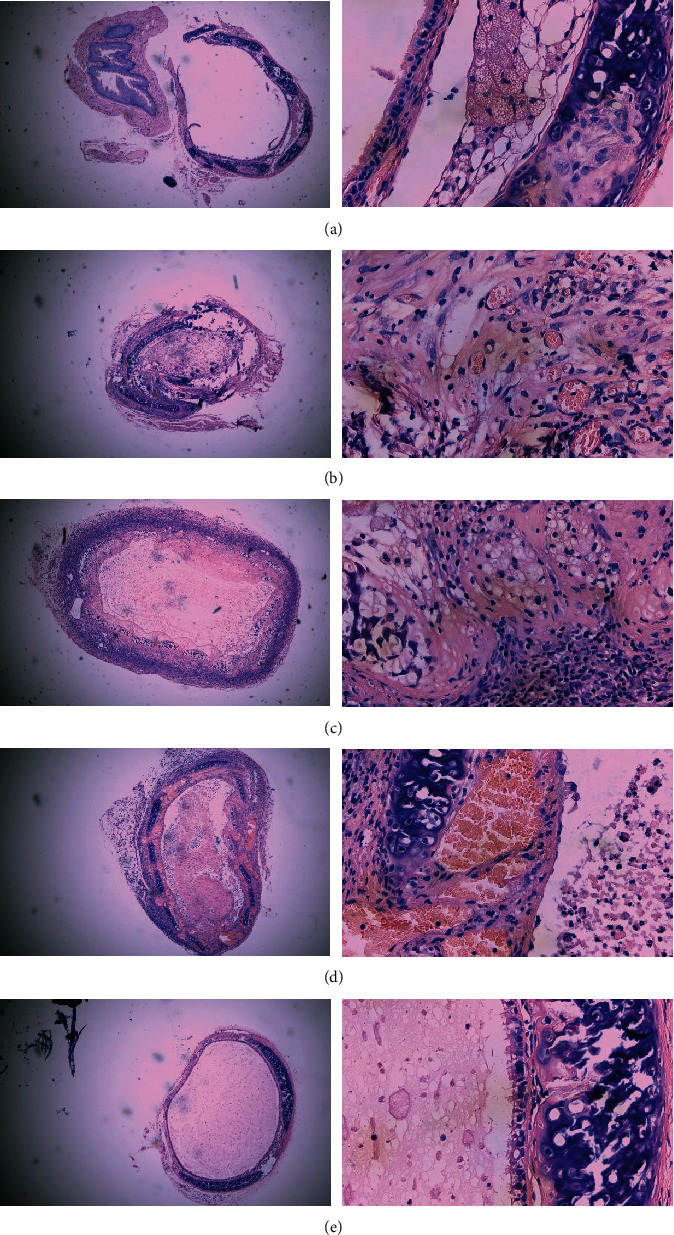
Pathologic histological changes of tracheal graft in different groups of mouse heterotopic tracheal transplantation model (hematoxylin-eosin staining, the magnification of the left side of figure was 40, and the magnification of the right side of figure was 400). (a) Isograft group; (b) allograft group; (c) allograft+azithromycin group; (d) allograft+tacrolimus group; (e) allograft+tacrolimus+azithromycin group.

No tracheal occlusion occurred in grafts of the isograft group. The rates of tracheal occlusion of the allograft group, allograft+tacrolimus group, allograft+azithromycin group, and allograft+tacrolimus+azithromycin group were 72.17 ± 4.66%, 40.33 ± 3.00%, 38.50 ± 2.08%, and 23.33 ± 3.24%, respectively. There were significant differences between the 4 groups (*p* < 0.001). Tacrolimus and azithromycin alone significantly reduced the occlusion rate of the transplanted trachea in allograft mouse heterotopic tracheal transplantation model (*p* = 0.0012 and 0.0011, respectively). The effect of tacrolimus and azithromycin in combination was superior to tacrolimus and azithromycin alone (*p* = 0.0239 and 0.0002, respectively) (Figure 4).

### 3.5. The Protein Expression of Cytokines and MMPs in Tracheal Graft of Different Groups of Mouse Heterotopic Tracheal Transplantation Model Determined by Western Blot Analysis

**Figure 5:**
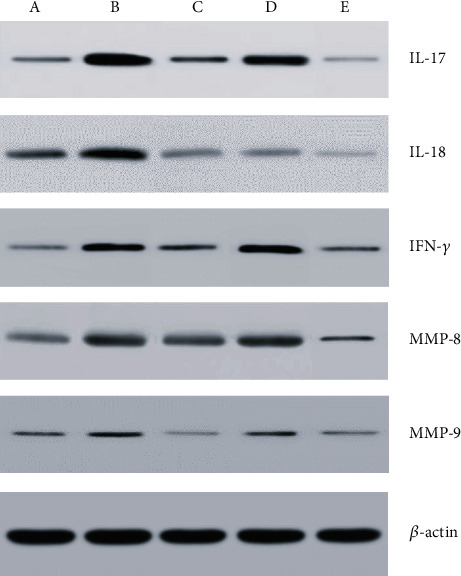
The protein expression of IL-17, IL-18, IFN-*γ*, MMP-8, and MMP-9 in tracheal graft of different groups of mouse heterotopic tracheal transplantation model determined by western blot analysis. (a) Isograft group; (b) allograft group; (c) allograft+ tacrolimus group; (d) allograft+azithromycin group; (e) allograft+tacrolimus+azithromycin group.

The protein expressions of IL-17, IL-18, IFN-*γ*, MMP-8, and MMP-9 in tracheal graft of the allograft group were higher than those of the isograft group. The protein expressions of IL-17, IL-18, IFN-*γ*, MMP-8, and MMP-9 in tracheal graft of the allograft+tacrolimus group were lower than those of the allograft group. The protein expression of IL-18 and MMP-9 in tracheal graft of allograft+azithromycin was lower than that of the allograft group. The protein expressions of IL-17, IL-18, IFN-*γ*, MMP-8, and MMP-9 in tracheal graft of the allograft+tacrolimus+azithromycin group were lower than those of the allograft group (Figure 5).

### 3.6. Serum Cytokines and MMPs of Different Groups of Mouse Heterotopic Tracheal Transplantation Model Determined by ELISA

Serum protein expressions of IL-17 (*p* = 0.0043), IL-18 (*p* = 0.0051), MMP-8 (*p* = 0.0464), and MMP-9 (*p* = 0.0262) were significantly increased in the allograft group compared to the isograft group. Serum protein expressions of IL-18 (*p* = 0.0351) and MMP-9 (*p* = 0.0482) were significantly decreased in the allograft+azithromycin group compared to the allograft group. Serum protein expressions of IL-17 (*p* = 0.0141), IL-18 (*p* = 0.0073), and MMP-9 (*p* = 0.0404) were significantly decreased in the allograft+tacrolimus group compared to the allograft group. Serum protein expressions of IL-17 (*p* = 0.0017), IL-18 (*p* = 0.0036), IFN-*γ* (*p* = 0.0102), and MMP-9 (*p* = 0.0194) were significantly decreased in the allograft+tacrolimus+azithromycin group compared to the allograft group (Table 1).

## 4. Discussion

Bronchiolitis obliterans syndrome (BOS), the clinical manifestation of OB, represented the most important cause of long-term allograft dysfunction and mortality after lung transplantation. Enhanced immunosuppression was an effective treatment for slowing or reversing the progression of OB in a certain part of patients; however, other patients were ineffective. Exploring the molecular mechanism of OB was conducive to prevention and treatment of BOS [23]. IL-18 was a cytokine which can stimulate various cell types to perform pleiotropic functions. IL-18 induced IFN-*γ* in natural killer cells and CD4 T helper 1 lymphocytes. Also, IL-18 also modulated Th2 and Th17 cell responses, as well as the activity of CD8 cytotoxic cells and neutrophils, in a host microenvironment-dependent manner. IL-18 was involved with varied of diseases, such as allergy, kidney diseases, metabolic disorders, cancer, and organ transplantation. IL-18 could be a target in immunotherapeutic or prophylactic interventions in infectious and noninfectious diseases [8, 24, 25]. In the present study, the roles of IL-18 in postlung transplantation OB were revealed.

Mouse heterotopic tracheal transplantation was the animal model used in this study. Heterotopic tracheal allografts in small rodents had been shown to share many characteristics with the development of obliterative bronchiolitis (OB) in the clinic and therefore provided a suitable animal model for the study of OB. The model facilitated the examination of the pathogenesis of the disease and the elucidation of the cellular and molecular mechanisms involved in its development. The model provided a less technically demanding alternative to whole lung transplantation in small rodents and should lead to a speedier identification of new treatments that might prevent the development of posttransplantation OB in the clinic [26–28]. Our experimental results showed that the expression of IL-18 mRNA and protein of the siRNA-IL-18 group was lower than that of the control group, and the luminal obliteration rates of the siRNA-IL-18 group were significantly lower than those of the control group.

Meanwhile, we also found that mRNA expressions of IFN-*γ*, EMMPRIN, MMP-8, and MMP-9 of tracheal graft were reduced in the siRNA-IL-18 group compared to the control group. A study showed that IL-18 induced airway hyperresponsiveness and lung fibrosis by IFN-*γ* and IL-13 production. IL-18 nasally administered acted on memory type T helper 1 cells and induced airway hyperresponsiveness and inflammation, which was characterized by peribronchial infiltration with neutrophils and eosinophils. Also, this administration induced lung fibrosis. Th1 cells were stimulated with antigen (Ag) and IL-18 and then secrete several cytokines, including IFN-*γ* and bronchogenic cytokine IL-13. Neutralization of IL-13 or IFN-*γ* during Ag plus IL-18 challenges inhibited the combination of eosinophilic infiltration, lung fibrosis, and periostin deposition or the combination of neutrophilic infiltration and airway hyperresponsiveness, respectively [29]. Another study defined the spectrum of inflammatory, parenchymal, airway, and vascular alterations that are induced by pulmonary IL-18. IL-18 induced type 1, type 2, and type 17 cytokines with IFN-*γ*-inhibiting macrophage, lymphocyte, and eosinophil accumulation while stimulating alveolar destruction and genes associated with cell cytotoxicity and IL-13 and IL-17A inducing mucus metaplasia, airway fibrosis, and vascular remodeling [30]. IL-18/IFN-*γ* axis might play a crucial role in process of airway inflammation and fibrosis.

Regulative effects of IL-18 on expression of MMP-8 and MMP-9 could be mediated by extracellular matrix metalloproteinase inducer (EMMPRIN). The cross-regulatory interaction between IL-18 and EMMPRIN had been investigated in human monocytes. Expression levels of IL-18 were positively associated with expression levels of EMMPRIN in monocytes. In vitro, the expression of EMMPRIN was increased in monocytes treated with IL-18; the secretion of IL-18 increased in monocytes treated with EMMPRIN. The downregulation of EMMPRIN expression by RNAi small interfering RNA reduced monocyte secretion of both IL-18 and MMP-9 [31]. MMP-9 was an important effector molecule in tissue remodeling during the occurrence process of OB. IL-18 initiated release of MMP-9 from peripheral blood mononuclear cells [32]. Cross-regulation between IL-18 and EMMPRIN in monocytes might amplify the inflammatory cascade and be responsible for increased monocytic MMP-9 and MMP-8 serum levels, contributing to occurrence and development of OB.

In this study, the effect of tacrolimus and azithromycin on OB-related molecules was investigated. Both tacrolimus and azithromycin significantly reduced the occlusion rate of the transplanted trachea in allograft mouse heterotopic tracheal transplantation model. Combined effect of tacrolimus and azithromycin was superior to tacrolimus and azithromycin alone. Known OB-related molecules, IL-17, IFN-*γ*, MMP-8, and MMP-9, were demonstrated to be the effector molecules of tacrolimus and azithromycin in anti-OB therapy. IL-18 expression was reduced after tacrolimus and azithromycin treatment and might be a novel target molecule for OB.

In this study, tacrolimus at 1 mg/kg/day dose significantly reduced the rates of tracheal occlusion in mouse heterotopic tracheal transplantation model. According to a murine pharmacokinetic study, an ideal dose of 1 mg/kg/day of tacrolimus intraperitoneally produced blood trough levels in the human therapeutic range (5-12 ng/ml) [33]. A prospective, randomized international trial in lung transplantation assessed the efficacy and safety of two immunosuppression, tacrolimus and cyclosporine, to prevent BOS. The results displayed that tacrolimus use was found to be associated with a significantly reduced risk for BOS grade ≥ 1 at 3 years compared with cyclosporine despite a similar rate of acute rejection [34]. According to a Cochrane systematic review involved a total of 413 adult lung transplant recipients, tacrolimus seemed to be significantly superior to cyclosporin regarding the incidence of BOS [35]. The effects of various immunosuppressants, tacrolimus and sirolimus, on airway epithelium were examined in an animal model which Brown Norway donor trachea were heterotopically transplanted into the greater omentum of Lewis. Both tacrolimus and sirolimus inhibited peritracheal inflammatory infiltration and luminal obliteration. Tacrolimus treatment had a more effective protection of the luminal epithelial coverage than sirolimus treatment [36]. Based on the above researches, tacrolimus seemed to be the most effective immunosuppressant for OB treatment.

Azithromycin had become a standard of care in therapy of BOS following lung transplantation. The alleviate effect of azithromycin on tracheal occlusion was observed in mouse heterotopic tracheal transplantation model in the present study. Although the dosage per kilogram body weight of azithromycin in mouse model was 4 times the normal dose used in patients, it was a low-dose administration (15 mg/kg/day) of azithromycin to our mouse heterotopic tracheal transplantation model according to previous literature [37] and the effects of azithromycin on tracheal occlusion were shown. The expression of IL-17 and MMP-9 in BOS could be attenuated by azithromycin. MMP-9 bronchoalveolar lavage levels increase in airway neutrophilia and BOS. IL-17 played a role in lung allograft rejection [37]. A study was to determine the effect of azithromycin on the airway epithelial barrier both in an in vitro model and in patients with asthma. Primary human bronchial epithelial cells (HBECs) were grown at air liquid interface (ALI) and challenged using lipopolysaccharides from Pseudomonas aeruginosa. Azithromycin was added at various stages and barrier integrity assessed using transepithelial electrical resistance (TEER) and permeability to FITC-dextran. MMP-9 levels were measured using ELISA. Azithromycin enhanced barrier integrity (TEER/FITC-dextran), increased thickness, and suppressed mucin production and MMP-9 release during the formation of a normal epithelial barrier in vitro. To provide translation of the findings, 10 patients with moderate-severe asthma were recruited and received 250 mg azithromycin once a day for 6 weeks. Bronchial biopsies taken pre- and postazithromycin treatment did not show evidence of increased epithelial barrier thickness or decreased mucin production. Similarly, bronchial wash samples did not show reduced MMP-9 levels [38].

In conclusion, IL-18 was a novel participant in the occurrence and development of OB, and its mechanism was that IL-18 played the positive regulatory role of IFN-*γ*, MMP-8, and MMP-9. IL-18 also could be the target for both tacrolimus and azithromycin treatment in OB therapy. Further clinical research on the role of IL-18 in prediction of OB incidence and treatment outcome should be made; also, dose effect relationship between these drugs and IL-18 remains to be revealed. The present study would contribute to precise prevention and treatment of OB after lung transplantation.

## Figures and Tables

**Table 1:** Serum cytokines and MMPs of different groups of mouse heterotopic tracheal transplantation model.

	Isograft group (*n* = 12)	Allograft group (*n* = 12)	Allograft+azithromycin group (*n* = 12)	Allograft+tacrolimus group (*n* = 12)	Allograft+tacrolimus+azithromycin group (*n* = 12)	*p ^a^*	*p* ^b^	*p ^c^*	*p* ^d^
IL-17 (*μ*g/L)	31.92 ± 1.16	37.77 ± 1.38	34.34 ± 0.99	32.80 ± 0.96	30.81 ± 0.96	0.0043	0.0531	0.0141	0.0017
IL-18 (*μ*g/L)	95.66 ± 3.21	115.3 ± 4.12	103.4 ± 2.43	98.42 ± 2.79	96.77 ± 2.75	0.0051	0.0351	0.0073	0.0036
IFN-r (ng/L)	68.04 ± 2.99	75.51 ± 2.72	70.03 ± 2.58	69.07 ± 2.60	65.73 ± 2.69	0.0606	0.1124	0.0783	0.0102
MMP-8 (*μ*g/L)	170.1 ± 8.89	196.3 ± 4.39	181.0 ± 7.93	171.6 ± 8.98	180.0 ± 7.48	0.0464	0.0999	0.0567	0.0690
MMP-9 (*μ*g/L)	44.28 ± 3.18	59.42 ± 2.58	51.71 ± 2.38	51.45 ± 2.24	50.81 ± 2.42	0.0262	0.0482	0.0404	0.0194

*p* ^a^: statistical differences between isograft group and allograft group, *p*^b^: statistical differences between allograft group and allograft+azithromycin group, *p*^c^: statistical differences between allograft group and allograft+tacrolimus group, and *p*^d^: statistical differences between allograft group and allograft+tacrolimus+azithromycin group.

## Data Availability

No data were used to support this study.
